# A Two-Stage Location Problem with Order Solved Using a Lagrangian Algorithm and Stochastic Programming for a Potential Use in COVID-19 Vaccination Based on Sensor-Related Data

**DOI:** 10.3390/s21165352

**Published:** 2021-08-09

**Authors:** Xavier Cabezas, Sergio García, Carlos Martin-Barreiro, Erwin Delgado, Víctor Leiva

**Affiliations:** 1 Faculty of Natural Sciences and Mathematics, Universidad Politécnica ESPOL, Guayaquil 090902, Ecuador; joxacabe@espol.edu.ec (X.C.); cmmartin@espol.edu.ec (C.M.-B.); edelgado@espol.edu.ec (E.D.); 2 School of Mathematics, The University of Edinburgh, Scotland EH9 3FD, UK; sergio.garcia-quiles@ed.ac.uk; 3 Faculty of Engineering, Universidad Espíritu Santo, Samborondón 0901952, Ecuador; 4 School of Industrial Engineering, Pontificia Universidad Católica de Valparaíso, Valparaíso 2362807, Chile

**Keywords:** heuristic algorithm, Lagrangian and semi-Lagrangian relaxations, mathematical programming, SARS-Cov2, sensing and data extraction, simple plant and uncapacitated facility location problems, XPRESS software

## Abstract

Healthcare service centers must be sited in strategic locations that meet the immediate needs of patients. The current situation due to the COVID-19 pandemic makes this problem particularly relevant. Assume that each center corresponds to an assigned place for vaccination and that each center uses one or more vaccine brands/laboratories. Then, each patient could choose a center instead of another, because she/he may prefer the vaccine from a more reliable laboratory. This defines an order of preference that might depend on each patient who may not want to be vaccinated in a center where there are only her/his non-preferred vaccine brands. In countries where the vaccination process is considered successful, the order assigned by each patient to the vaccination centers is defined by incentives that local governments give to their population. These same incentives for foreign citizens are seen as a strategic decision to generate income from tourism. The simple plant/center location problem (SPLP) is a combinatorial approach that has been extensively studied. However, a less-known natural extension of it with order (SPLPO) has not been explored in the same depth. In this case, the size of the instances that can be solved is limited. The SPLPO considers an order of preference that patients have over a set of facilities to meet their demands. This order adds a new set of constraints in its formulation that increases the complexity of the problem to obtain an optimal solution. In this paper, we propose a new two-stage stochastic formulation for the SPLPO (2S-SPLPO) that mimics the mentioned pandemic situation, where the order of preference is treated as a random vector. We carry out computational experiments on simulated 2S-SPLPO instances to evaluate the performance of the new proposal. We apply an algorithm based on Lagrangian relaxation that has been shown to be efficient for large instances of the SPLPO. A potential application of this new algorithm to COVID-19 vaccination is discussed and explored based on sensor-related data. Two further algorithms are proposed to store the patient’s records in a data warehouse and generate 2S-SPLPO instances using sensors.

## 1. Introduction

The process of making decisions based on a solution to a problem, within the context of mathematical modeling, can take several stages. In each of these stages, events are defined in a such way that may be dependent on each other and on the variables that they define.

The use of random variables in the above-mentioned form of decision making may be described within a stochastic programming context. Then, the model can be represented as a sequential decision-making process, where the variables of one stage that are unknown at first are part of the input parameters of the subsequent stage.

A stochastic programming problem is a mathematical formulation with constraints where some of its components can be represented by random variables. If the problem has decisions that have to be made after the uncertainty is resolved (realization), it takes the name of the resource program [1]. The values of the random variables are known after carrying out an experiment, and decisions can be made at a first stage and at a second stage, before and after the experiment, respectively. In addition, each stage can contain a succession of decisions and they can be made over different periods of time. In this case, a multi-state stochastic programming problem should be formulated. Thus, we can say that a stochastic programming problem is multi-state if the decisions depend on realizations that occur over time. Since its introduction in the seminal papers by Dantzig [2] and Beale [3], the stochastic framework has been widely studied and applied in diverse areas, such as transportation [4], inventory management [5], drug supply [6], and industrial processes [7], among others.

Another example of the above framework is the so-called simple plant location problem, SPLP in short [8]. In the SPLP, given a set of possible locations, we must decide where to open facilities and then distribute resources demanded by certain customers/patients, minimizing the costs involved. Note that the SPLP is a combinatorial approach, where the mathematical model can be viewed as one of two stages that tries to answer two questions: (i) what facilities should be opened, and (ii) how should these facilities be located in such a way that the customer demand is met? The second question must be answered after the facilities (unknown at the beginning) are stated, that is, after the variables defined at this stage for such a decision are revealed by conducting an experiment that considers a possible future state of a second stage. Therefore, it is logical to think that it is necessary to define different forms of this state of nature that represent possible future scenarios such that the effect of uncertainty is minimized. Described in this way, the SPLP is known as a two-stage stochastic simple plan location problem, 2S-SPLP in short.

The 2S-SPLP has been widely studied, and its solution methods have been shown to be efficient. As mentioned in [1], it is important to consider that, taking advantage of the structure of the problem, the 2S-SPLP is especially beneficial in stochastic mathematical programming, and it is the main focus of the algorithmic work in this area. Here, the concept of structure refers to any characteristic in the mathematical formulation that can be used to provide a solution. One of the most-used procedures for stochastic mathematical programming that involves integer variables is the so-called integer L-shape method proposed in [9]. This method is a branch-cut algorithm that uses the L-shape that the coefficient matrix has in the stochastic formulation of the 2S-SPLP and solves subproblems obtained by the Benders decomposition. For details of this and other similar procedures, see [1,10]. Lagrangian relaxation (LR) is a less-used approach, but a full description of different applications can be found in the references previously cited. Some gradient (subgradient)-based methods, such as those used in LR, were applied over other algorithms to speed up the search for the optimum in discrete problems; see [11] as an example.

A less-known extension of the SPLP is when an order of preference, that customers have over a set of facilities to meet their demand, is considered. The SPLP with order is denoted by SPLPO in short [12,13], with the size of the instances in the SPLPO that can be solved being limited. Such an order incorporates a new set of constraints in its formulation that increases the difficulty to obtain an optimal solution. To the best of our knowledge, the use of order with two stages in the SPLP based on stochastic programming has not been considered until now. We use an algorithm that incorporates a heuristic component to handle the complexity that is added when orders of preference are included.

The motivation for our study comes from a problem related to healthcare services, where service centers must be sited in strategic locations to meet the immediate needs of patients. The current situation due to the COVID-19 pandemic makes this problem particularly relevant [14]. Let us assume that each center corresponds to an assigned place for vaccination and that each center uses one or more vaccine brands from different laboratories. Then, each patient could choose one center instead of another, because she/he may prefer the vaccine from a laboratory to be considered more reliable. This defines a ranking (order of preference) which will not necessarily be given for all of them, since a patient may not want to be vaccinated in a center where there are only non-preferred vaccine brands. In countries where the vaccination process is considered successful, the order of preference assigned by each patient to the vaccination centers is defined by incentives that local governments give to their population. These same incentives for foreign citizens are seen as a strategic decision to generate income from tourism.

The main objective of this work is to solve the problem of large instances based on a more general version of the 2S-SPLP, which we call the two-stage stochastic simple plant location problem with order (2S-SPLPO). Our secondary objective is to show a potential use of the 2S-SPLPO in COVID-19 vaccination based on sensor-related data. Indeed, we provide an algorithm that stores patient’s service records in a central data warehouse using sensors. We conduct computational experiments on simulated instances to assess the performance of the 2S-SPLPO.

In the 2S-SPLPO, the customers have preferences over the set of facilities that can serve them. We consider the possibility that the order of preference given by patients is partial, that is, a patient may want to be served by only a subset of all possible facilities that may be open in some locations. In the stochastic formulation that we propose for the 2S-SPLPO, together with the variables defined to decide in which locations to open facilities, the preferences (partial or complete) are considered random variables. To solve the 2S-SPLPO, we use the accelerated dual ascent (ADA) algorithm proposed in [12,13], which solves large instances of the non-stochastic version of the SPLPO. Our algorithm is based on that given in [15] for the SPLP. The ADA algorithm uses LR and semi-Lagrangian relaxation (SLR) in an iterative procedure to approximate the solution. The SLR is a relatively new idea that exploits the structure of problems by relaxing a direction of a split equality constraint. The ADA algorithm tries to solve a Lagrangian dual (LD) problem with a subgradient method (SGM) as a first step. Then, the results of the first step are used in a second step to solve a semi-Lagrangian dual (SLD) problem with a dual ascent method (DAM). At last, a heuristic procedure is applied to accelerate the search of the optimum in a third step. For a complete description of the SGM, the reader is referred to [16,17,18,19,20,21], whereas for a description of the SLR, the reader is referred to [15,22] Applying heuristic procedures is a common practice in optimization, but without guarantee of reaching the optimum. Some creative algorithms help to finding sufficiently good solutions [23]. In the next sections, we shows how the ADA algorithm produces good solutions, comparing them to the exact solutions given by the XPRESS software.

Other two potential applications of the our approach can be seen in [24,25], where one of them is found in business-to-consumer (B2C) e-commerce. As pointed out in [25], e-commerce has logistical advantages over traditional B2C. However, although costs can be reduced considerably, due to the use of technology, the problem of distributing the products demanded to patients from centers exists still. Sometimes, patients prefer to pick up the products from these centers and, therefore, may have preferences about where to do it, as the distance to travel or travel times can influence the problem’s variables. Thus, the aim of the B2C e-commerce organizations lies in finding the best way to locate the distribution centers, reducing the logistics costs related to this process and also considering the order of preference of the centers. These applications proposed in [24,25] are complemented with the potential application in COVID-19 vaccination based on the sensor-related data proposed in the present investigation.

The rest of the article is distributed as follows. Section 2 provides background on stochastic programming. Section 3 and Section 4 define the SPLPO and 2S-SPLPO, respectively. In Section 5, a description of the three steps that form the ADA algorithm is provided. In Section 6, we introduce two sensor-based algorithms that store patients’ service records in a central data warehouse and that generate 2S-SPLPO instances, respectively. In Section 7, we report the results of computational experiments performed on large instances. Finally, in Section 8, some conclusions and recommendations for future studies on this topic are outlined.

## 2. Two-Stage Stochastic Linear Programming

In this section, we provide the background of stochastic programming, with MP standing for mathematical program. In MP 1, $E_{\xi }$ denotes the mathematical expectation on the random vector values ξ, whereas $c\in R^{n_{1}}$, $b\in R^{m_{1}}$, $A\in R^{m_{1}\times n_{1}}$, and $W\in R^{m_{2}\times n_{2}}$ are known vectors and matrices.

Let $\Omega =\left\{\;\omega_{1},\ldots ,\omega_{|\Omega |}\;\right\}$ be a finite sample space of some experiment. Let ξ be a random variable over the elements of Ω with values ξ(ω). For simplicity, we call any ξ(ω) the scenario ω. In a stochastic program, the scenarios can be seen as states of the nature since all the unknown information could depend on them. In addition, we assume a discrete probability function expressed as

(1) $$p\left(\xi \right)=P\left(\xi =\xi \left(\omega \right)\right)=\alpha^{\omega }.$$

The vectors $y\in R^{n_{1}}$ and $x\in R^{n_{2}}$ are the decision variables of the first and second stages, respectively. Therefore, y can be considered the decisions to be made under uncertainty, and x the corrective decisions made when uncertainty is revealed. Furthermore, $q\left(\omega \right)\in R^{n_{2}}$, $h\left(\omega \right)\in R^{m_{2}}$ and $T\left(\omega \right)\in R^{m_{2}\times n_{1}}$ are vectors with known components after the realization of ω, with T(ω) being usually called the technological matrix and W the fixed resource matrix. Let $T_{i}\left(\omega \right)$ be the *i*-th row of T(ω). A random vector $\chi \left(\omega \right)={\left[q\left(\omega \right),h\left(\omega \right),T_{1}\left(\omega \right),\ldots ,T_{m_{2}}\left(\omega \right)\right]}^{⊤}\in R^{n_{2}+m_{2}+\left(m_{2}\times n_{1}\right)}$ is implicitly defined in MP 1, whose formulation was originally stated in [2,3].

**MP 1** Two-stage stochastic mathematical programming formulation with fixed resources.
$$\begin{array}{ccc} \min\;Z & = & c^{⊤}y+E_{\xi }\left[\min q{\left(\omega \right)}^{⊤}x\right]\\ \mathrm{subject}\mathrm{to}\;Ay & = & b,\\ T(\omega )y+Wx & = & h(\omega ),\\ y & \geq & 0,\\ x & \geq & 0. \end{array}$$


## 3. Simple Plant Location Problem with Order

In this section, we define the SPLPO. Let I={1,…m} be a set of customers, and let J={1,…n} be a set of possible sites where facilities can be opened. Let $J^{i}$ be a subset of *J* defined for each customer *i*, with $|J^{i}|=n_{i}$ and $\bar{J^{i}}=J\setminus J^{i}$. Unit costs $c_{ij}\geq 0$ are considered to supply the demand of customer *i* from facility *j*, and fixed costs $f_{j}\geq 0$ to open a facility at location *j*. We say that *k* is *i*-worse than *j* if the customer *i* prefers facility *j* rather than *k*, which is denoted by $k{<}_{i}j$. We define $W_{ij}=\left\{k\in J|k{<}_{i}j\right\}$ as the set of facilities *k* strictly *i*-worse than *j*, with its complement being denoted as $\bar{W_{ij}}$ and $W_{ij}\cup \left\{j\right\}$ as $W_{ij}^{\prime }$. Let $x_{ij}$ be a decision variable that represents the fraction of the demand required by the customer *i* and covered by facility *j*. Let $y_{j}$ be a binary variable such that $y_{j}=1$ if a facility is open at the location *j*, and $y_{j}=0$ otherwise. We assume that each customer *i* classifies her/his favorite facility $j\in J^{i}$ with a number $p_{ij}\in \left\{1,\ldots ,n_{i}\right\}$, where 1 and $n_{i}$ are the most and least preferred, respectively. Additionally, for all $j\in \bar{J^{i}}$, we have that $p_{ij}=n_{i}+1$. Under these conditions, the linear program for the SPLPO is given by MP 2.

**MP 2** SPLPO mathematical programming formulation.
(2)$$\begin{array}{ccc} \min\;Z & = & \sum_{i\in I}\sum_{j\in J}c_{ij}x_{ij}+\sum_{j\in J}f_{j}y_{j}, \end{array}$$
(3)$$\begin{array}{ccc} \mathrm{subject}\mathrm{to}\;\sum_{j\in J}x_{ij} & = & 1,\;\forall i\in I, \end{array}$$
(4)$$\begin{array}{ccc} x_{ij} & \leq & y_{j},\;\forall i\in I,j\in J, \end{array}$$
(5)$$\begin{array}{ccc} \sum_{k\in \bar{W_{ij}}}x_{ik} & \geq & y_{j},\;\forall i\in I,j\in J^{i},\; \end{array}$$
(6)$$\begin{array}{ccc} x_{ij} & \geq & 0,\;\forall i\in I,j\in J, \end{array}$$
(7)$$\begin{array}{ccc} y_{j} & \in & \{0,1\},\;\forall j\in J. \end{array}$$


The statement expressed in (2) says that the program minimizes a cost function related to opening a facility covering the demand. Equalities presented in (3) ensure that customer *i* is supplied by exactly facility *j*, called assignment constraints. Constraints given in (4) ensure that if customer *i* is supplied by facility *j*, then *j* must be opened, which are often called varying upper bounds. Inequalities stated in (5) model the customers’ orders of preference [26]. Since no capacities are considered and the model minimizes the number of open facilities *y*, the demand of customer *i* can always be covered completely by one single facility. Therefore, we can guarantee that there is an optimal solution with the values of the variables $x_{ij}$ belonging to {0,1}, even though this is not specified in the constraints; see inequalities given in (6). The family of inequalities presented in (7) makes the binary nature of the variable $y_{j}$ explicit. Note that if it is more favorable to open an installation that does not belong to a particular subset $J^{i}$, customer *i* can be served by any open installation, since $n_{i}+1$ is the worst classification given for any *j*.

## 4. Stochastic Formulation for the SPLPO

In this section, we define the 2S-SPLPO. In the context of the SPLPO, consider the experiment of asking all customers i∈I to rate their preferred facilities j∈J. Such as that defined in MP 1, we call each result of this experiment ω and the set of all of them the sample space Ω. Recall the random variable ξ with values ξ(ω) over the events {ω}∈Ω called the scenario ω. Once again, we assume that this random variable has the discrete probability function stated in (1). Under this setting and the mentioned probability function, a mathematical program for the 2S-SPLPO (complete or partial) can be written as in MP 3.

**MP 3** 2S-SPLPO mathematical programming formulation.
(8)$$\begin{array}{ccc} \min\;Z & = & \sum_{j\in J}f_{j}y_{j}+\sum_{\omega \in \Omega }\alpha^{\omega }\sum_{i\in I}\sum_{j\in J}c_{ij}x_{ij}^{\omega } \end{array}$$
(9)$$\begin{array}{ccc} \mathrm{subject}\mathrm{to}\;\sum_{j\in J}x_{ij}^{\omega } & = & 1,\;\forall i\in I,\omega \in \Omega , \end{array}$$
(10)$$\begin{array}{ccc} x_{ij}^{\omega } & \leq & y_{j},\;\forall i\in I,j\in J,\omega \in \Omega , \end{array}$$
(11)$$\begin{array}{ccc} \sum_{k\in \bar{W_{ij}^{\omega }}}x_{ik}^{\omega } & \geq & y_{j},\;\forall i\in I,j\in J^{i},\omega \in \Omega , \end{array}$$
(12)$$\begin{array}{ccc} x_{ij}^{\omega } & \geq & 0,\;\forall i\in I,j\in J,\omega \in \Omega , \end{array}$$
(13)$$\begin{array}{ccc} y_{j} & \in & \{0,1\},\;\forall j\in J. \end{array}$$


**Figure 1:**
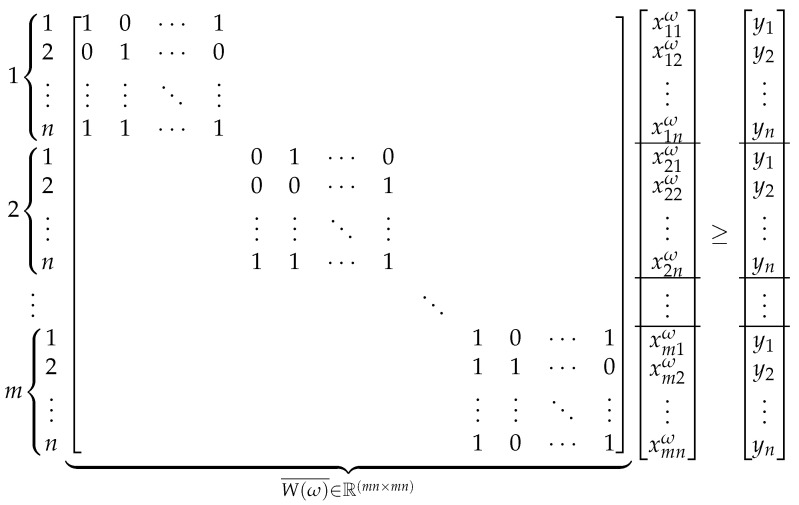
An example of the matrix $\bar{W(\omega )}$.

Note that the mathematical expressions defined in (8)–(13) of MP 3 have a similar interpretation as those stated in MP 2 for each scenario ω. The second component established in (8) represents the expected value of the distribution cost over the scenarios. Consider that the first decision stage to open a facility is given by binary variable *y* and the distribution of the demand is given by *x* as the second stage. Regarding the family of constraints formulated as in (11) of MP 3, each scenario ω is represented by a {0,1}-matrix W(ω) ($\bar{W(\omega )}$) of dimension (mn×mn), where each row ij has a one for each element of $W_{ij}^{\omega }$ ($\bar{W_{ij}^{\omega }}$) and a zero for elements of its complement; see Figure 1. Furthermore, Ω is a finite set but it can have a big cardinality. For example, if we consider no partial preferences and since n<+∞, each row of W(ω) ($\bar{W(\omega )}$) can be given in $2^{n}$ different ways. Thus, there are ${\left(2^{n}\right)}^{mn}=2^{mn^{2}}$ possible different matrices W(ω) ($\bar{W(\omega )}$).

Note that the stochastic program formulated in MP 3 has no fixed resources since the coefficient matrix $\bar{W(\omega )}$ given by the sets $\bar{W_{ij}^{\omega }}$ with the second-stage variables $x_{ij}^{\omega }$ is random, that is, it depends on ω.

## 5. A Lagrangian Algorithm for the 2S-SPLPO

**Figure 2:**
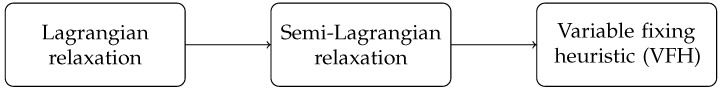
Scheme of steps in the ADA algorithm.

In this section, we state a Lagrangian algorithm for the 2S-SPLPO. As noted in MP 3, since the mathematical formulation of the 2S-SPLPO has similar features to its non-stochastic version (SPLPO), with obvious differences due to the presence of multiple random scenarios, we suggest applying the ADA algorithm. Figure 2 shows the scheme of the ADA algorithm, which includes three modules that we have broken down into internal steps in a very general way. The SLR theory and some important results, which are exposed in the description of the ADA algorithm, can be found in [15,22], where this algorithm was introduced. The main idea of the SLR approach is to relax a direction of an equality constraint in the Lagrangian sense, keeping the other one without relaxing, and then to take advantage of the mathematical properties of the resulting programming. Next, we detail each of the steps of the ADA algorithm summarized in Figure 2.

Step 1: Lagrangian relaxation

In MP 3, we relax two families of constraints: those of assignment stated in (9) and those of customer preference defined in (11), obtaining the Lagrangian program given by

(14) $$\begin{array}{ccc} \mathrm{LR}(\mu^{\omega },\lambda^{\omega }) & := & \min_{\left(x^{\omega },y\right)}\;\sum_{j}f_{j}y_{j}+\sum_{\omega }\alpha^{\omega }\sum_{i}\sum_{j}c_{ij}x_{ij}^{\omega }\\ & & +\sum_{\omega }\sum_{i}\mu_{i}^{\omega }(1-\sum_{j}x_{ij}^{\omega })+\sum_{\omega }\sum_{i}\sum_{j}\lambda_{ij}^{\omega }(y_{j}-\sum_{k\in \bar{W_{ij}^{\omega }}}x_{ik}^{\omega })\\ & = & \min_{\left(x^{\omega },y\right)}\sum_{\omega }\sum_{i}\sum_{j}(\alpha^{\omega }c_{ij}x_{ij}^{\omega }-\mu_{i}x_{ij}^{\omega }-\lambda_{ij}^{\omega }\sum_{k\in \bar{W_{ij}^{\omega }}}x_{ik}^{\omega })\\ & & +\sum_{j}(f_{j}y_{j}+\sum_{\omega }\sum_{i}\lambda_{ij}^{\omega }y_{j})+\sum_{\omega }\sum_{i}\mu_{i}^{\omega },\\ \mathrm{subject}\mathrm{to}: & & (10),(12),(13). \end{array}$$

In the case where a partial order is considered, for all $j\in \bar{J^{i}}$, the corresponding terms $\lambda_{ij}^{\omega }\sum_{k\in \bar{W_{ij}^{\omega }}}x_{ik}^{\omega }$ and $\sum_{\omega }\sum_{i}\lambda_{ij}^{\omega }y_{j}$ vanish in the objective function defined in (14).

As before, we are assuming that customer *i* ranks for each scenario her/his favorite facility $j\in J^{i}$ with a number $p_{ij}^{\omega }\in \left\{1,\ldots ,n_{i}\right\}$, where 1 and $n_{i}$ are the most and least preferred, respectively. Since in $\sum_{\omega }\sum_{i}\sum_{j\in J^{i}}\lambda_{ij}^{\omega }\sum_{k\in \bar{W_{ij}^{\omega }}}x_{ik}^{\omega }$ each $\lambda_{ij}^{\omega }$ is multiplied by a sum of $x_{ij}^{\omega }$ variables with $p_{ij}^{\omega }$ terms corresponding to $k\geq_{i}j$, then each $x_{ij}^{\omega }$ is multiplied by a sum of $\lambda_{ik}^{\omega }$ with $n_{i}-p_{ij}^{\omega }+1$ terms corresponding to $k\leq_{i}j$. Thus, we can check the following:

(15) $$\sum_{\omega }\sum_{i}\sum_{j\in J^{i}}\lambda_{ij}^{\omega }\sum_{k\in \bar{W_{ij}^{\omega }}}x_{ik}^{\omega }=\sum_{\omega }\sum_{i}\sum_{j\in J^{i}}x_{ij}^{\omega }\sum_{k\in W_{ij}^{{}^{\prime }\omega }}\lambda_{ik}^{\omega },$$

where $|W_{ij}^{{}^{\prime }\omega }|=n_{i}-p_{ij}^{\omega }+1.$

By using the expression given in (15), the objective function defined in (14) can be rewritten as

(16) $$\begin{array}{ccc} \mathrm{LR}(\mu^{\omega },\lambda^{\omega }) & := & \min_{\left(x^{\omega },y\right)}\;\sum_{j}[\sum_{\omega }\sum_{i}(\alpha^{\omega }c_{ij}-\mu_{i}^{\omega }-\Lambda_{ij}^{\omega })x_{ij}^{\omega }\\ & & +(f_{j}+\sum_{\omega }\sum_{i}\lambda_{ij}^{\omega })y_{j}]+\sum_{\omega }\sum_{i}\mu_{i}^{\omega },\\ \mathrm{subject}\mathrm{to}: & & (10),(12),(13), \end{array}$$

where $\Lambda_{ij}^{\omega }=\sum_{k\in W_{ij}^{{}^{\prime }\omega }}\lambda_{ik}^{\omega },$ with $|W_{ij}^{{}^{\prime }\omega }|=n_{i}-p_{ij}^{\omega }+1.$

Similar to its non-stochastic version, with and without order, SPLP and SPLPO, respectively, the problem stated in (16), with multipliers $\mu^{\omega }$ and $\lambda^{\omega }$ fixed, can be easily solved by considering that

$$y_{j}=\left\{\begin{array}{cc} 1, & \mathrm{if}\sum_{i}\min\left(0,\alpha^{\omega }c_{ij}-\mu_{i}^{\omega }-\Lambda_{ij}^{\omega }\right)+\left(f_{j}+\sum_{\omega }\sum_{i}\lambda_{ij}^{\omega }\right)<0,\\ 0, & \mathrm{otherwise}, \end{array}\right.$$

and

$$x_{ij}^{\omega }=\left\{\begin{array}{cc} 1, & \mathrm{if}y_{j}=1\mathrm{and}\alpha^{\omega }c_{ij}-\mu_{i}^{\omega }-\Lambda_{ij}^{\omega }<0,\\ 0, & \mathrm{otherwise}. \end{array}\right.$$

The problem stated in (16) has the integrality property, that is, its optimal value is equal to the standard linear relaxation LP (2S-SPLPO), which corresponds to the original problem without the integrality constraints. The ADA algorithm tries to solve the LD problem related to (16) established as

$$\mathrm{LD}\left(\mu^{\omega },\lambda^{\omega }\right):=\max_{\left(\mu^{\omega },\lambda^{\omega }\right)}\mathrm{LR}\left(\mu^{\omega },\lambda^{\omega }\right),$$

with a SGM, due to the fact that its objective function is a piecewise linear concave function of vector multipliers $\mu^{\omega }$ and $\lambda^{\omega }$ over its domain. Hence, after some iterations, the multipliers obtained are used as a starting point for the second step. The SGM requires multiple times to solve the problem given in (16), but as mentioned, it is simple to solve; see Algorithm 1, and for its step in line see (12) and also [19,20,21]. 


**Algorithm 1: **Subgradient method.

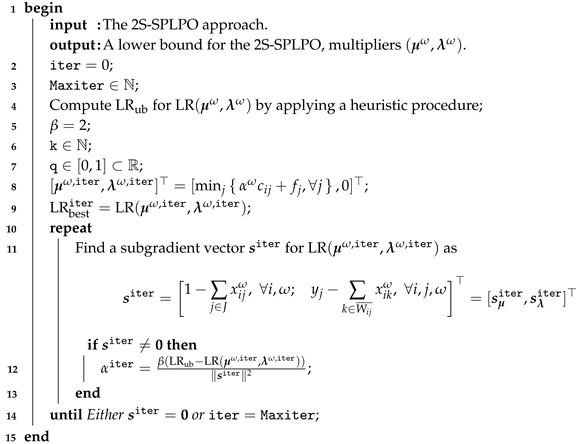




   It is possible to prove that the vector computed in step 11 of Algorithm 1 is a subgradient of the Lagrangian problem. In our experiments, we obtain better results when $\mu_{i}^{\omega ,0}=\min_{j}\left\{\;\alpha^{\omega }c_{ij}+f_{j}\;\right\}$, for all i∈I and ω∈Ω instead $\mu^{\omega ,0}=0$. In step 4 of Algorithm 1, we set ${\mathrm{LR}}_{\mathrm{ub}}$ by applying an upper bound heuristic for the 2S-SPLP (H2S); see Algorithm 2. First, it opens a facility with the lowest operating cost for all customers. Then, for those facilities that are not open yet, the procedure compares and chooses for each customer the most preferred facility between it and its previously assigned supplier. Consequently, the new cost is saved and the new open facility has the lowest operating cost. This is repeated until no more facilities are available.
**Algorithm 2:** Upper bound heuristic for the 2S-SPLP.
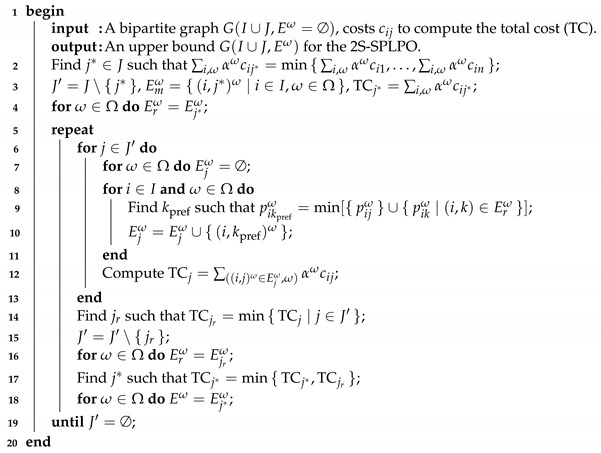

Step 2: Semi-Lagrangian relaxation

We start with a brief description of the SLR. Let the problem (P) of mathematical program be stated as

(17) $$(P):=\min_{x}\left\{\;f\left(x\right)=c^{⊤}x|Ax=b,x\in X\;\right\},$$

with (i) the set X containing integrality constraints; and (ii) $A\in R^{m\times n}$ and rational components $b\in R^{m},c\in R^{n}$ being non-negative.

After splitting Ax=b into Ax≥b and Ax≤b, inequality Ax≥b is relaxed with a vector of multipliers λ≥0. Then, the SLR problem related to (P) is expressed as

$$\mathrm{SLR}\left(\lambda \right):=\min_{x}\left\{f\left(x\right)+\lambda \left(b-Ax\right)|Ax\leq b,x\in X\right\},$$

with its SLD being given by $\mathrm{SLD}\left(\lambda \right):=\max_{\lambda \geq 0}\mathrm{SLR}\left(\lambda \right)$.

The following theorems summarizes the basic properties derived from the definition of the SLR.

**Theorem** **1** ([15]). *Let* (P)* be a problem as defined in* (17) *under the same conditions. Then, we have the following:*

(i) SLR(λ)*is concave, non-decreasing on its domain, and*b−Ax*is a subgradient at the point *λ.
(ii) There is an interval $\left[\lambda^{*},+\infty \right)$ where for each multiplier, we obtain the optimal solution of SLR(λ).
(iii) *LP(P)≤LD(λ)≤SLD(λ)=(P), that is, SLR(λ) closes the duality gap.*

In MP 3, we semi-relax the assignment constraints defined in (9). Thus, we obtain the program formulated as

$$\begin{array}{cc} \mathrm{SLR}(\gamma^{\omega }) & :=\min_{\left(x^{\omega },y\right)}\;\sum_{\omega }\sum_{i}\sum_{j}c_{ij}x_{ij}^{\omega }+\sum_{j}f_{j}y_{j}+\sum_{\omega }\sum_{i}\gamma_{i}^{\omega }(1-\sum_{j}x_{ij}^{\omega })\\ \; & =\min_{\left(x^{\omega },y\right)}\;\sum_{j}(\sum_{\omega }\sum_{i}(\alpha^{\omega }c_{ij}-\gamma_{i}^{\omega })x_{ij}^{\omega }+f_{j}y_{j})+\sum_{\omega }\sum_{i}\gamma_{i}^{\omega }, \end{array}$$

subjectto(10),(11),(12),(13), and $\sum_{j\in J}x_{ij}^{\omega }\leq 1$, ∀i∈I,ω∈Ω.

The corresponding dual problem is stated as $\mathrm{SLD}\left(\gamma^{\omega }\right):=\max_{\gamma^{\omega }}\mathrm{LR}\left(\gamma^{\omega }\right).$ We follow the same idea given in [15], in the context of the SLR applied to the uncapacitated facility location problem, to restrict our search for the vector of multipliers $\gamma^{\omega }$ in $\mathrm{SLD}(\gamma^{\omega })$.

**Theorem** **2.** Let $\mathrm{SLR}(\gamma^{\omega })$ and $c_{i}^{\left(\omega \right)}=\max_{j}\left\{\alpha^{\omega }c_{ij}+f_{j}\right\}$, $c^{\omega }=\left(c_{1}^{\left(\omega \right)},\ldots ,c_{m}^{\left(\omega \right)}\right)$ be the maximal cost for costumer i associated with facility j and the vector of these costs, respectively. Then, we obtain $\gamma^{\omega }\in Q=\left[\gamma^{*},+\infty \right)$ if $\gamma^{\omega }\geq c^{\omega }$.

**Proof.**  If $\sum_{j\in J}x_{ij}^{\omega }=1$ for all *i* and ω∈Ω, the SLR closes the duality gap. By hypothesis, $c_{i}^{\left(\omega \right)}-\gamma_{i}^{\omega }\leq 0$. If we choose $j^{\prime }$ such that $c_{i}^{\left(\omega \right)}=\alpha^{\omega }c_{ij^{\prime }}+f_{j^{\prime }}$, then $\left(\alpha^{\omega }c_{ij^{\prime }}-\gamma_{i}^{\omega }\right)+f_{j^{\prime }}\leq 0$. This inequality is true for any *j* since $j^{\prime }$ gives the maximum among all $\alpha^{\omega }c_{ij}+f_{j}$. Therefore, the event $\sum_{j\in J}x_{ij}^{\omega }=0$ cannot happen at an optimal solution since it is always possible to set $x_{ij}^{\omega }=1$ and $y_{j}=1$ for all *i* and *j*, meeting all the constraints.    □

**Theorem** **3.** *Let $\mathrm{SLR}(\gamma^{\omega })$. For each i∈I and ω∈Ω, let $\alpha^{\omega }c_{i}^{\left(1\right)}\leq \cdots \leq \alpha^{\omega }c_{i}^{\left(n\right)}$ be the sorted cost $\alpha^{\omega }c_{ij}$. Then, if $\gamma^{\omega }<\alpha^{\omega }c^{1}$, $\gamma^{\omega }\notin Q$.*

**Proof.**  By hypothesis, $\alpha^{\omega }c_{i}^{\left(1\right)}-\gamma_{i}^{\omega }>0$. Then, we have that $\alpha^{\omega }c_{i}^{\left(j\right)}-\gamma_{i}^{\omega }>0$ for all *j* since we are minimizing at the optimal solution $x_{ij}^{\omega }=0$ for all *j*. Therefore, the event $\sum_{j\in J}x_{ij}=1$ cannot happen at an optimal solution and $\gamma^{\omega }\notin Q$.    □

Theorems 2 and 3 limit the search of $\gamma^{\omega }$ to $B=\{\gamma^{\omega }|\alpha^{\omega }c^{\left(1\right)}<\gamma \leq c^{\omega }\}$. We use the multipliers $\mu^{\omega }$ obtained in the previous step as starting point in this step by setting $\gamma^{\omega }=\mu^{\omega }$. Then, based on Theorems 2 and 3, we solve the LD problem by increasing the components of $\gamma^{\omega }$ in each iteration of a DAM that requires solving $\mathrm{SLR}(\gamma^{\omega })$ many times; see Algorithm 3. However, this problem is harder than $\mathrm{LR}(\mu^{\omega },\lambda^{\omega })$. The DAM algorithm employs the following:

- By using the sorted costs $\alpha^{\omega }c_{i}^{\left(1\right)}\leq \cdots \leq \alpha^{\omega }c_{i}^{\left(n=|J|\right)}$, each component $\gamma_{i}^{\omega }$ of $\gamma^{\omega }$ can be either in an interval of the form $\left(\alpha^{\omega }c_{i}^{\left(j\right)},\alpha^{\omega }c_{i}^{\left(j+1\right)}\right]$ or out of it. For the first case, there are infinite values of $\gamma_{i}^{\omega }$ that can belong to a single interval $\left(\alpha^{\omega }c_{i}^{\left(j\right)},\alpha^{\omega }c^{\left(j+1\right)}\right]$. Each one of them has the same effect in the solution of $\mathrm{SLR}(\gamma^{\omega })$ since only a change from $y_{j}=1$ to $y_{j+1}=1$ and $x_{ij}^{\omega }=1$ to $x_{i,j+1}^{\omega }=1$ modifies the solution.
- We just need a single $\gamma_{i}^{\omega }$ representative of the intervals. As we get closer to solving the SLR, the values of the components of $\gamma^{\omega }$ increase. Hence, solving the SLR becomes more and more difficult. Then, it is always convenient to choose a $\gamma_{i}^{\omega }\in I_{i}$ being as small as possible, that is, at an ε distance from the lower bound of an interval.

**Algorithm 3: **Dual ascent method.

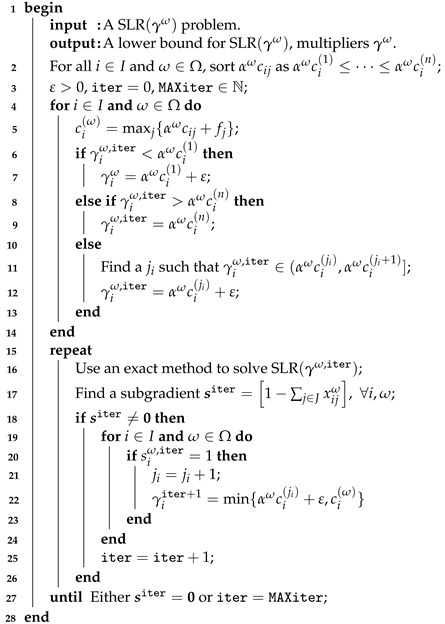

Step 3: Variable fixing heuristic

A heuristic method is used to speed up the search process to the optimum. In a run of the DAM, the procedure chooses by a cost criteria, from the solution given, a subset corresponding to a percentage ps×100% of the best variables $y_{j}$, such that $y_{j}=1$. These *y* are fixed to solve the 2S-SPLPO in the next iteration by an exact method. The procedure is described in Algorithm 4. In addition, the procedures described above are assembled in the ADA method stated in Algorithm 5.


**Algorithm 4: **Variable fixing heuristic.

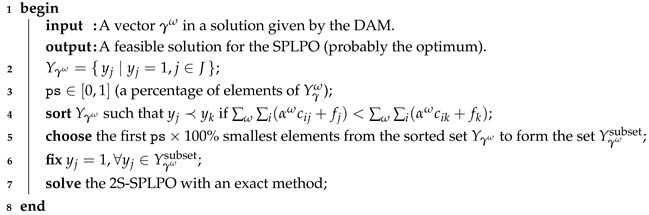





**Algorithm 5: **Accelerated dual ascent algorithm.

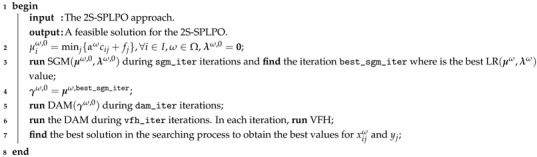




## 6. Sensing Patients’ and Simulated Data for the Proposed Methodology

In this section, we propose an algorithm that allows us to update patients’ records and store them in a central data warehouse. Consider COVID-19 vaccination centers. Patients go to the different care points based on an order of preference. At each point, there is a local repository that stores the data of the patients who were treated at that point. However, the data must be centralized in a single database for persistent storage purposes and also for later analysis. With a certain periodicity (for example, at the end of the day), a sensor located at each local repository sends the data related to the care of each of the patients to the central warehouse. It is important to keep in mind that each vaccination center usually stores the local data for one day of care. In a general scenario, Algorithm 6 permits us to centralize, in a warehouse, the data obtained when patients are served in the facilities. In addition, to simulate data and solve instances of the 2S-SPLPO, we propose Algorithm 7 which sensorizes the database.
**Algorithm 6: **Approach for updating patients’ records in a central data warehouse using sensors.1:Clear the local data repository of each facility at the beginning of the operations.2:Store a data record in the local repository for each patient and for each facility.3:Send the patients’ records to a central data warehouse by a sensor located at the local data repository of the facility at the end of the operations.4:Store the patient’s records for all facilities permanently in the central data warehouse.5:Use the central warehouse for any data analysis.

**Algorithm 7: **Approach to generate and solve instances of the 2S-SPLPO with a sensor.
1:Generate data for the second scenario with the simulation algorithm.2:Build an instance of the 2S-SPLPO with the data simulated in the previous step for the data taken in the first scenario.3:Store the instance in the database.4:Detect the instance of the 2S-SPLPO with a sensor in the database and dynamically build the mathematical model.5:Call the ADA algorithm to solve the mathematical model with the sensor.6:Receive the mathematical model and solve it with the ADA algorithm.7:Store the output of the ADA algorithm in the database and generate tables with the execution results.


## 7. Computational Experiments

In this section, we show the results of computational experiments obtained after applying the ADA algorithm to a set of 2S-SPLPO (complete preferences) with two scenarios. Problems with one scenario were taken from [27], which are based on the Beasley OR-Library [28]. The second scenario was randomly generated. In order to simulate data and solve instances of the 2S-SPLPO, we use Algorithm 7 which sensorizes the database.

The experiments were carried out on a PC with Intel*®* Xeon*®* 3.40 GHz processor and 16 GB of RAM under a Windows*®* 10 operating system. All problems were solved with FICO XPRESS*®* version 8.0.

Table 1 shows the parameter settings for all steps of the ADA algorithm, which were set after an extensive experimental process. We observe that there is a positive relation between the number of iterations of the SGM and the size of the problem, with sgm_iter being set at a value greater than or equal to *n*. Due to the difficult to solve $\mathrm{SLR}(\gamma^{\omega }$) in the DAM, sgm_iter was set with a value less than a 10% of *n*.

The names of the problems in Table 2 and Table 3 use the nomenclature as given in the following example: [10a][7550]_[2], where [10a]: The second scenario is 10% different from the first scenario a; [7550]: m=75 and n=50; and [2]: Problem 2 with first scenario a. In the literature on the topic (for example, in p. 145, Section 4 of [27]), the instances shown in Table 3 are considered large. Note that, although the times in many cases increase as the size of the problem increases, in some cases, this behavior is not detected. For example, in Table 3, the ADA algorithm solves the problem 100b10075_1 in 3531 seconds. However, in a larger instance, such as 100a150100_1, the time is less (1253 seconds). Since the ADA algorithm is a heuristic, the running times can vary, even on the same problem, due to the random component that the VFH has. Therefore, we cannot affirm the exponential growth of the running times.

The results show that the ADA algorithm performs very well in all cases. Indeed, the optimum was found in most of them. Note that when the percentage of changed preferences was 10% (first six instances in Table 2), the ADA algorithm was not able to improve the XPRESS times. Nevertheless, in the remaining cases, the times were improved considerably with a couple of exceptions. From Table 3, observe that the ADA algorithm attains the optimal solution in most of the cases in much less time, with the exception of the problem 100c150100_1, where the time was greater than that obtained by the XPRESS software. The last of the four groups corresponds to the same problems in the third group of Table 3 (m=150, n=100). Nonetheless, they were tested with a different parameter sgm_iter=140. For all these six cases, the ADA algorithm improves the running times and bounds. The column headers in Table 2 and Table 3 mean the following:

- Prob: Name of the problem.
- Opt: Optimal value of the problem.
- LP(P): Linear relaxation value for a problem (P).
- GAP${}_{X}=\left(\mathrm{Opt}-\mathrm{LP}\right)/\mathrm{Opt}\times 100\%$: Relative gap between Opt and LP of a problem by using the XPRESS software.
- t: Time in seconds
- H2Sub: Best upper bound with the H2S.
- y: Number of opened facilities.
- SGMlb: Lower bound with the SGM.
- ADAub: Best upper bound with the ADA algorithm.
- DAMlb: Lower bound with the DAM (without the VFH).
- GAP${}_{V}=\left(\mathrm{bestUB}-\mathrm{LB}\right)/\mathrm{bestUB}\times 100\%$: Relative gap between the best upper bound and lower bound of a problem by using the DAM with VHF.
- Tt: Total time in seconds.
- imp t: $(\left(t_{\mathrm{opt}}-t_{\mathrm{ADA}}\right)/t_{\mathrm{opt}})\times 100\%$
- GAP: ((ADAub−Opt)/Opt)×100%.

## 8. Conclusions, Limitations, and Future Research

Our work was motivated from a problem related to healthcare services, where service centers must be sited in strategic locations that meet the immediate needs of patients. The COVID-19 pandemic makes this problem particularly relevant [29]. We have provided an algorithm that stores patients’ records in a central data warehouse using sensors. This allowed us to propose a potential application of our new algorithm to COVID-19 vaccination based on sensor-related data. Specifically, in our investigation, we have proposed a Lagrangian-based approach for the 2S-SPLPO, called the ADA algorithm. It has three steps that are related and work consecutively to create a heuristic procedure that takes advantage of the structure of the problem.

In summary, this paper reported the following findings:

(i) Formulations for SPLPO and 2S-SPLPO with partial preferences were proposed.
(ii) Lagrangian and semi-Lagrangian structures for the 2S-SPLPO with partial preferences were introduced.
(iii) A theoretical analysis of properties of the Lagrangian and semi-Lagrangian structures for the 2S-SPLPO was presented to combine them in a procedure that approximates its solution.
(iv) Theorems 2 and 3 were stated as extensions for the 2S-SPLPO of those given in [15] for the uncapacitated facility location problem.
(v) To the best of our knowledge, there have been no algorithms proposed to solve the stochastic version of the SPLPO. The proposed algorithm is a novel approach that uses a relatively new optimization technique known as semi-Lagrangian relaxation.
(vi) The computational experiments suggested that the ADA algorithm performed satisfactorily on large instances in both search of the optimum and execution time.
(vii) Possible applications in real cases are described in the context of the COVID-19 vaccination process and B2C e-commerce.

Some limitations of our study are the following:

(i) Since the last step of the ADA algorithm is a heuristic, the optimal is not guaranteed.
(ii) The ADA algorithm is studied in the context of the 2S-SPLPO, so its use is limited to it. We suggest studying the same ideas in other location problems.
(iii) A cost–benefit evaluation should be carried out in the use of the proposed algorithm to answer the following question: is the improvement in execution times worth it, with respect to the savings obtained in the value of the objective function?
(iv) Studies and experiments with deeper parameter settings and more scenarios must be performed on larger instances.
(v) We suggest carrying out a computational experiment that allows us to determine the parameter values of the mathematical optimization model from which it is necessary to design and implement a heuristic or metaheuristic algorithm. High computational complexity problems can be solved using the heuristic approach. Some examples of this can be found in [30,31,32].

The authors are working on these and other issues associated with the present investigation. The corresponding findings are expected to be reported in future works.

## Figures and Tables

**Table 1:** Parameter settings of the ADA algorithm for the indicated method. Source: the authors.

*m*	*n*	SGM				DAM		VFH	
		sgm_iter	β	k	q	dam_iter	ε	vfh_iter	ps
75	50	50	2	30	0.005	3	0.01	2	0.25
100	75	100	2	30	0.005	7	0.01	2	0.25
125	100	170	2	30	0.005	10	0.01	2	0.25
150	100	170	2	30	0.005	12	0.01	2	0.25

**Table 2:** ADA algorithm applied to the 2S-SPLPO with two scenarios (m=75, n=50). Source: the authors.

Prob	XPRESS				H2S			SGM		DAM with VFH				ADA		
	Opt	LP	GAP${}_{X}$	t	H2Sub	y	t	SGMlb	t	ADAub	DAMlb	GAP${}_{V}$	t	Tt	imp t	GAP
10a7550_1	1,648,853	1,200,745	27%	67	1,787,955	1	0	840,831	7	1,698,650	1,279,041	25%	60	67	0%	3.02%
10a7550_2	1,602,647	1,193,378	26%	48	1,738,071	3	0	763,664	6	1,602,647	1,286,736	20%	50	56	−17%	0.00%
10b7550_1	1,226,979	901,051	27%	48	1,335,244	10	0	334,589	5	1,226,979	953,159	22%	57	62	−28%	0.00%
10b7550_2	1,263,465	906,280	28%	53	1,299,259	10	0	372,981	7	1,271,641	970,770	24%	70	77	−46%	0.65%
10c7550_1	1,290,291	900,033	30%	46	1,388,280	9	0	591,938	7	1,290,291	971,785	25%	62	69	−48%	0.00%
10c7550_2	1,248,312	876,053	30%	47	1,365,717	13	0	557,308	6	1,248,312	936,483	25%	65	71	−51%	0.00%
25a7550_1	1,670,734	1,199,273	28%	119	1,761,050	5	0	830,624	6	1,670,734	1,284,052	23%	53	59	50%	0.00%
25a7550_2	1,606,360	1,195,482	26%	67	1,730,389	3	0	770,870	7	1,606,360	1,269,850	21%	53	60	11%	0.00%
25b7550_1	1,256,755	892,106	29%	58	1,329,801	11	0	360,026	6	1,259,217	947,822	25%	61	67	−15%	0.20%
25b7550_2	1,324,578	907,308	32%	153	1,348,842	7	0	380,921	6	1,324,578	988,646	25%	98	104	32%	0.00%
25c7550_1	1,364,933	907,957	33%	166	1,413,489	9	0	579,362	8	1,373,542	978,857	29%	96	104	37%	0.63%
25c7550_2	1,271,029	873,435	31%	84	1,291,327	13	0	578,313	7	1,271,029	958,058	25%	77	84	0%	0.00%
50a7550_1	1,689,428	1,212,042	28%	113	1,778,238	6	0	804,221	7	1,689,428	1,302,236	23%	60	67	41%	0.00%
50a7550_2	1,637,084	1,196,440	27%	86	1,744,372	5	0	760,256	6	1,637,084	1,273,267	22%	51	58	33%	0.00%
50b7550_1	1,331,207	900,654	32%	165	1,388,062	12	0	294,443	7	1,331,207	1,247,451	6%	381	388	−136%	0.00%
50b7550_2	1,307,706	896,181	31%	182	1,364,303	11	0	305,591	7	1,307,706	990,369	24%	94	101	45%	0.00%
50c7550_1	1,347,482	911,370	32%	145	1,446,345	12	0	565,472	6	1,347,482	976,268	28%	77	84	42%	0.00%
50c7550_2	1,287,982	878,852	32%	112	1,439,845	17	0	546,735	7	1,299,952	952,655	27%	71	78	30%	0.93%
100a7550_1	1,787,955	1,208,842	32%	267	1,787,955	1	0	824,731	8	1,787,955	1,293,098	28%	89	97	64%	0.00%
100a7550_2	1,683,058	1,204,739	28%	194	1737,,924	5	0	827,668	7	1,720,482	1,268,026	26%	66	72	63%	2.22%
100b7550_1	1,451,139	935,113	36%	363	1,496,648	6	6	311,032	6	1,453,678	989,340	32%	86	92	75%	0.17%
100b7550_2	1,400,184	916,167	35%	271	1,449,128	9	0	358,259	7	1,400,205	1,013,362	28%	106	113	58%	0.00%
100c7550_1	1,360,674	920,342	32%	169	1,430,298	9	0	562,495	8	1,360,674	998,681	27%	106	114	32%	0.00%
100c7550_2	1,402,514	918,696	34%	196	1,462,990	16	0	570,058	8	1,404,147	989,592	30%	102	110	44%	0.12%

**Table 3:** ADA algorithm applied to the 2S-SPLPO with two scenarios for m∈{100,125,150} and n∈{75,100}. Source: the authors.

Prob	XPRESS				H2S			SGM		DAM with VFH				ADA		
	Opt	LP	GAP${}_{X}$	t	H2Sub	y	t	SGMlb	t	ADAub	DAMlb	GAP${}_{V}$	t	Tt	imp t	GAP
100a10075_1	2,469,439	1,811,464	27%	561	2,476,632	1	1	1,644,492	31	2,476,632	1,978,083	20%	265	296	47%	0.29%
100a10075_2	2,458,870	1,805,025	27%	685	2,476,632	1	1	1,627,278	32	2,458,870	1,971,011	20%	237	270	61%	0.00%
100b10075_1	2,132,719	1,364,985	36%	17,935	2,270,467	6	1	1,112,051	37	2,132,719	1,558,555	27%	3531	3568	80%	0.00%
100b10075_2	2,163,818	1,367,450	37%	27679	2,218,215	7	1	1,160,875	43	2,163,818	1,564,516	28%	4380	4422	84%	0.00%
100c10075_1	1,978,807	1,271,848	36%	14,835	2,072,702	6	1	1,052,860	49	1,988,903	1,496,210	25%	1027	1076	93%	0.51%
100c10075_2	1,987,757	1,261,290	37%	11,567	2,118,928	9	1	1,066,388	51	1,987,757	1,452,609	27%	3152	3202	72%	0.00%
100a125100_1	3,070,535	2,416,518	21%	918	3,070,535	1	2	2,237,118	93	3,070,535	2,619,571	15%	573	666	27%	0.00%
100a125100_2	3,070,535	2,388,054	22%	1088	3,070,535	1	1	2,239,726	106	3,070,535	2,587,669	16%	702	808	26%	0.00%
100b125100_1	2,800,573	1,815,018	35%	53,666	2,850,413	5	2	1,601,481	118	2,850,413	2,078,979	27%	20,837	20,955	61%	1.78%
100b125100_2	2,820,883	1,820,001	35%	78,669	3,019,740	4	1	1,592,305	143	2,820,883	2,016,632	29%	8230	8373	89%	0.00%
100c125100_1	2,702,169	1,698,737	37%	239,967	2,866,218	10	2	1,488,148	157	2,702,169	1,990,068	26%	23,717	23,874	90%	0.00%
100c125100_2	2,716,252	1,705,149	37%	204,007	2,829,945	5	1	1,477,796	168	2,717,597	2,007,831	26%	27,442	27,610	86%	0.05%
100a150100_1	3,768,087	2,924,250	22%	1735	3,768,087	1	1	2699949	109	3,768,087	3239975	14%	905	1014	42%	0.00%
100a150100_2	3,768,087	2,918,397	23%	1819	3,768,087	1	1	2702231	111	3,768,087	3,251,719	14%	117	228	87%	0.00%
100b150100_1	3,412,417	2,179,897	36%	169,739	3,637,438	1	1	1,923,456	141	3,412,417	2,599,980	24%	21,111	21,252	87%	0.00%
100b150100_2	3,388,309	2,196,284	35%	69,508	3,637,438	1	2	1,924,300	169	3,388,309	2,679,093	21%	14792	14,962	78%	0.00%
100c150100_1	3,287,595	2,010,587	39%	502,354	3,413,288	4	2	1,768,341	185	3,413,288	N/A	N/A	N/A	N/A	N/A	3.82%
100c150100_2	3,229,424	2,012,045	38%	494,721	3,307,459	5	3	1,776,475	132	3,300,341	2,474,515	25%	119,700	119,832	76%	2.20%
100a150100_1	3,768,087	2,924,250	22%	1735	3,768,087	1	1	2,635,877	94	3,768,087	3,229,888	14%	1159	1253	28%	0.00%
100a150100_2	3,768,087	2,918,397	23%	1819	3,768,087	1	1	2,637,900	109	3,768,087	3,242,679	14%	1255	1364	25%	0.00%
100b150100_1	3,412,417	2,179,897	36%	169,739	3,637,438	1	2	1,876,775	147	3,445,585	2,607,746	24%	21,590	21,738	87%	0.97%
100b150100_2	3,388,309	2,196,284	35%	69,508	3,637,438	4	4	1,863,975	155	3,388,309	2,599,780	23%	8984	9139	87%	0.00%
100c150100_1	3,287,595	2,010,587	39%	502,354	3,413,288	4	2	1,750,243	179	3,288,348	2,457,997	25%	78,577	78756	84%	0.02%
100c150100_2	3,229,424	2,012,045	38%	494,721	3,307,459	5	3	1,718,731	185	3,230,261	2,470,331	24%	144,729	144,729	71%	0.03%

## Data Availability

The data analyzed are available upon request.
